# Enhancement of effector functions of anti-CD20 monoclonal antibody by increased afucosylation in CHO cell line through cell culture medium optimization

**DOI:** 10.1186/s43141-022-00421-5

**Published:** 2022-10-04

**Authors:** Bala Reddy Bheemareddy, Prakash Narayana Reddy, Kranthi Vemparala, Vijaya R. Dirisala

**Affiliations:** 1 R&D Division, Hetero Biopharma Limited, Jadcherla, Mahbubnagar, Telangana 509301 India; 2 Microbiology Department, Dr. V.S. Krishna Government Degree College (Autonomous), Visakhapatnam, Andhra Pradesh 530013 India; 3 Department of Biotechnology, Vignan’s Foundation for Science, Technology and Research (Deemed to be University), Guntur, Andhra Pradesh 522213 India

**Keywords:** Non-Hodgkin’s lymphoma, Anti-CD20 monoclonal antibody, Chinese hamster ovary cells, Afucosylation, Osmolality, Antibody-dependent cellular cytotoxicity

## Abstract

**Background:**

Recombinant therapeutic anti-CD20 monoclonal antibody (mAb) is used for the treatment of non-Hodgkin’s lymphoma, a common B cell lymphoma constituting 80% of all lymphomas. Anti-CD20 mAb contains an Fc-linked biantennary glycan. Although, anti-CD20 monoclonal antibodies are being increasingly used for immunotherapy, their efficacy is limited in a section of patients with drug resistance to immunotherapy. There is a need to improve the efficacy by increasing the effector functions, such as the antibody-dependent cellular cytotoxicity (ADCC) activity of anti-CD20 monoclonal antibodies.

**Results:**

We developed a simple and cost-effective approach to enhance ADCC effector activity in an in-house developed clone of anti-CD20 monoclonal antibody by increasing afucosylation in a new clone of Chinese Hamster Ovary (CHO) cells using 8X uridine, manganese, and galactose (UMG) to modulate the osmolality of the medium. The purified anti-CD20 monoclonal antibody from 8X UMG-containing medium showed a 2-fold increase in afucose content and 203% ADCC activity in comparison to control antibody.

**Conclusions:**

Our study reports enhanced ADCC activity by modulating afucosylation using osmolality by altering simple feed additives in the culture medium.

**Supplementary Information:**

The online version contains supplementary material available at 10.1186/s43141-022-00421-5.

## Background

Cancer is a complex disease which is a result of interplay of genetics, epigenetics, environment and lifestyle choices. Common cancer treatment options include chemotherapy, radiotherapy, surgery, and in some cases a joint therapy has shown encouraging results [1]. Non-Hodgkin’s lymphoma (NHL), a B cell lymphoma, is the most common type of lymphoma contributing to 80% of all lymphomas. Standard regimen options for B cell lymphoma treatment such as cyclophosphamide, doxorubicin hydrochloride (hydroxydaunorubicin), vincristine (Oncovin), and prednisone (CHOP) chemotherapy and radiation therapy are not effective in certain patient populations who have developed drug resistance [2, 3]. Recently, immunotherapy which is based on recombinant monoclonal antibodies (mAbs) has gained prominence as a viable treatment option in patients who do not respond to classical chemo and radiation therapies. Though immunotherapy with monoclonal antibodies (e.g., rituximab) has produced significant clinical benefits in B-cell lymphoma patients, there is a need to further increase the potency of immunotherapy to obtain durable clinical remission and to decrease mortality. For example, even though rituximab therapy is considered as remarkable treatment option for non-Hodgkin’s lymphoma, 30–50% of patients still do not respond clinically or display resistance to re-treatment [4]. The number of patients who relapse and are refractory to the immunotherapy is increasing alarmingly day by day. For these patients, there is no alternative therapy available currently and hence the risk of mortality is very high. Such limitations have stimulated efforts to improve the effectiveness of currently used anti-CD20 mAbs through antibody engineering [4].

The primary focus of antibody engineering is to increase the potency of the effector functions, which are the mechanisms of action for anti-CD20 mAbs such as antibody-dependent cellular cytotoxicity (ADCC), complement-dependent cytotoxicity (CDC), and apoptosis [5, 6]. ADCC is the most important mechanism of action for the efficacy of these antibodies in B cell lymphoma patients. In a mouse xenograft model of human B cell lymphoma, ADCC by anti-CD20 antibody was shown to be the major tumoricidal mechanism in vivo [4]. In ADCC, an antibody first binds to a cell-surface target antigen, then recruits immune effector cells that lyse the target cell. The affinity of the antibody to FcγRIIIa receptor and thereby its ADCC activity critically depends on the presence and composition of the N-linked glycans attached to the fragment crystallizable (Fc) portion of the antibody. For example, it is well established that the absence of core fucose leads to increased ADCC activity [7, 8].

A decrease in fucosylation at Asn297 (asparagine) has been shown to increase the antibody-binding affinity to CD16 (FcγRIIIa) on natural killer cells and ultimately increase ADCC potency [9, 10]. Hence, researchers have shown significant interest in lowering fucosylation to increase therapeutic antibody efficacy through approaches such as using glycoengineered antibodies in the clinic [11]. Currently, there are several new generations anti-CD20 mAbs engineered and/or modified to improve antitumor activity and Fc-binding affinity. These mAbs are advantageous over rituximab and are currently undergoing clinical investigation. However, although these glycol-engineered antibodies have higher potency for the targeted effector function, they display weaker potency for the remaining effector functions. Moreover, the process of glycoengineering is in itself very tedious, and demands expensive resources. Hence, there is a need to develop a more viable alternative process to increase the ADCC activity of anti-CD20 mAbs.

Modulation of antibody galactosylation in CHO cell cultures using feed additives is proven to increase the galactosylation. The routinely used feed additives are Uridine (U), Manganese chloride (M), and Galactose (G) in various strengths. As per the previous reports, 8X UMG increase the galactosylation up to 21% [12]. Manganese is the key ingredient shown to influence antibody fucosylation. Previously, a case study focusing on the glycan optimization and ADCC activity by using metal ions as media supplements had shown the linear correlation of manganese ion levels in cell culture media with non-fucosylated N-glycan species within a range from 0 to 40 μM. They demonstrated that the effect can be observed using manganese up to 500 μM, resulting in a reduction of fucosylation from 95 to 60% in 15 mL scale fed-batch cultivations. In contrast, high mannose type glycans were increased to only 15%. With this increase in the afucosylated glycan fraction, CD16-binding affinity of the mAb could be increased up to 350% (the linear correlation of CD16-binding affinity and afucosylated N-glycan levels) by increased manganese concentrations in cell culture media [10].

In this study, we have investigated, whether the level of afucosylation could be increased in an anti-CD20 monoclonal antibody by modulating the osmolality of the cell culture medium using 8X UMG with 250 μM Manganese chloride concentration, thereby improving the effector functions especially ADCC activity. We have developed a new clone of anti-CD20 mAb and attempted to enhance the ADCC activity by modulating afucosylation, which is a post-translational modification, in CHO cells.

## Methods

### CHO clone development

Dihydrofolate reductase (dhfr)-deficient Chinese hamster ovary (CHO) cells was adapted for growth in serum free medium and was used to construct stable high-producing cell line for the expression of anti-CD20 mAb. The primary amino acid sequence of anti-CD20 antibody was obtained using ESI-MS/MS and Edman sequencing of rituximab (Ristova®, Roche, India). The determined amino acid sequences of light chain (LC) and heavy chain (HC) of the antibody were back translated to a nucleotide sequence, which was optimized for codon usage and mRNA structure. Care was taken during DNA sequence assembly that no chain termination codons were present when translated in silico and the same reflected in in vivo expression. IDT Codon Optimization Tool for codon usage optimization by manually reassigning codon usage based on the frequency of each codon’s usage in the *Cricetulus griseus* (Chinese hamster) codon usage table. The resulting nucleotide sequences of both LC and HC were synthetized together with the signal sequences.

**Fig. 1:**
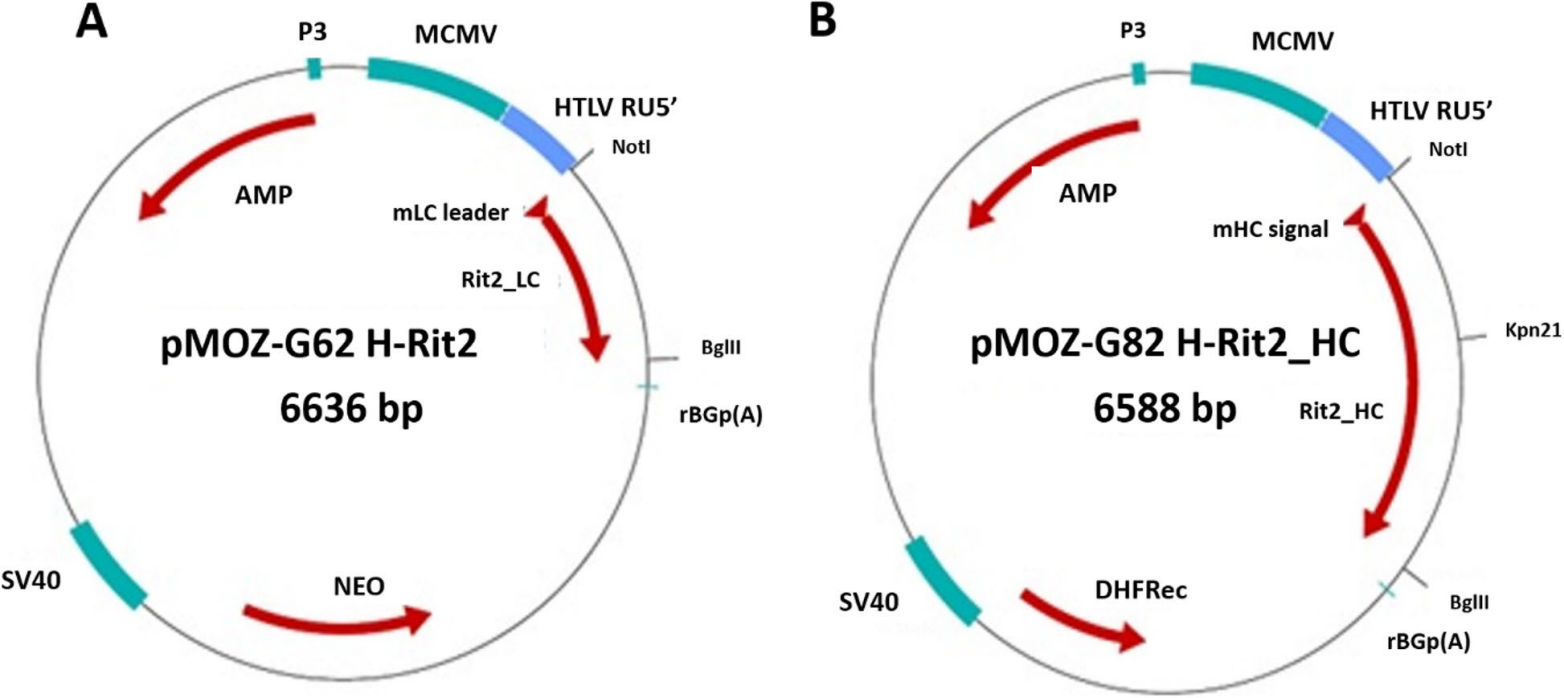
Diagrams showing the expression vectors with the cloned light (**A**) and heavy chain (**B**) sequences. pMOZ-G62 carry neomycin resistance (NEO) gene as a dominant selectable marker. pMOZ-G82 vector contains a copy of the mouse dihydrofolate reductase (DHFR) gene under control of the SV40 early promoter. DHFR confers methotrexate resistance in Chinese hamster ovary cells (CHO) which lack dihydrofolate reductase. Both vectors carry mouse cytomegalovirus (MCMV) promotor elements and rabbit beta globin polyadenylation (rBGp(A)) signals

The synthetic DNA fragments of the LC and HC of the antibody were linked to human T-cell leukemia virus (HTLV) 5′-UTR and cloned separately into the expression vectors pMOZ-G62 and pMOZ-G82, which are modified vectors based on pSV2-neo (ATCC®37149) and pSV2-dhfr (ATCC®37146) vectors respectively. Cloning and expression vectors were procured from EUGENEX Biotechnologies GmbH, Tägerwilen, Switzerland. Both the expression vectors consist of the same backbone, which includes a mouse cytomegalovirus promoter/enhancer element and a beta-globin polyadenylation site to drive the recombinant target gene, as well as a selection marker driven by a SV40 promoter cassette. While pMOZ-G62 contains the neomycin resistance gene as a dominant selectable marker, a recombinant hybrid dhfr gene is used as an amplifiable marker in pMOZ-G82. The resulting final expression plasmids were called pMOZ-G62 H-Rit2_LC for the expression of light chain and pMOZ-G82 H-Rit2_HC for the expression of the heavy chain of anti-CD20 mAb (Fig. 1). The CHO host cell line (DSMZ, Germany) was co-transfected with pMOZ-G62 H-Rit2_LC and pMOZ-G82 H-Rit2_HC using the lipofection transfection method. Anti-CD20 antibody production in transformed cells was discovered by dot ELISA experiment. The best performing primary clone (CHOSI Rit H3) was chosen for further sub-cloning by serial dilution on 96-well plates. As a final test, the best performing double clone was tested for cell growth and antibody expression using the shake flask-based fed-batch culture for 10 days.

### Expression of anti-CD20 monoclonal antibody with higher afucosylation

Two experiments were setup of which one served as the control experiment. The process parameters used for mAb production is listed in Table 1. Experiments were started with an initial seed culture of 0.5 × 10^6^ cells/mL in cell culture volume of 3750 mL comprising 10 mL/L U1 feed, 2.5 mL/L U2 feed, 20 mL/L CHO feed, 1 mL/L TE mix, and 1 mL/L lipid mix on day 0. From day 3 onwards, cells were fed with CHO feed, glucose, galactose, U1 and U2 feeds, TE mix, and lipid mix until day 11. Then, 50 μM of MnCl_2_ was supplemented on day 6 and 100 μM MnCl_2_ was supplemented on day 9 and day 11. The temperature shifts were carried out while keeping the pH and % dissolved oxygen (DO) constant throughout the culture period.

**Table 1:** Details of process parameters of the 5000 mL production bioreactor

Parameters	Set value	Duration
Temperature (°C)	37	Day 0 to 5
	34	Day 5 to 7
	32	Day 7 to 10
	30	Day 10 to 11
DO (%)	30	Day 0 to 11
pH	7.2	Day 0 to 11

To increase afucosylation in the test experiment, the culture medium was fed with additive 8X UMG. The composition of 8X UMG is 8 mM uridine, 250 μM manganese chloride, and 12.21 mM galactose, mixed in the culture medium and fed on day 6, 9, and 11 along with other feeds to modulate osmolality of the medium. The 8X UMG concentration that we finalized in our experiment were based on our own study trials and studies by other researchers [13, 14]. In the control experiment, the culture medium was maintained without 8X UMG. The rest of process parameters were maintained uniformly for both the experiments.

### Purification of anti-CD20 monoclonal antibody

#### Cell culture supernatant

The afucosylated anti-CD20 mAb containing cell culture harvest was subjected to purification to remove impurities. The harvest was centrifuged at 5000×*g* for 10–15 min in a fixed angle rotor at < 10 °C (Beckman Coulter, USA) and then the supernatant was collected and filtered using a 0.22 μm filter (Pall corporation, USA).

#### Affinity chromatography and low pH treatment (LpHT)

The filtered supernatant containing the antibody was purified by affinity chromatography using MabSelectSuRe LX resin (GE healthcare, USA). The operating parameters used for affinity chromatography are listed in Supplementary Table 1. The affinity eluent was collected as a single fraction (step elution) starting from 50 mAU ascending until 50 mAU descending. Viral inactivation has been carried out by employing low pH treatment (LpHT). The affinity eluent was kept under slow stirring condition using a magnetic stirrer and the pH was adjusted to 3.5 by slow addition of 2 M acetic acid. The eluent was incubated for 60 min under stirring. After incubation, the pH was re-adjusted to 7.2 by adding 1 M Tris base (pH 8.0).

#### Anti-CD20 mAb purification

The LpHT output was subjected to further purification by anion exchange (AEX) chromatography using Q-sepharose FF (GE healthcare, USA). The operating parameters used for this purification step are listed in Supplementary Table 1. The AEX flow-through fraction was collected from ascending 5 mAU to descending 10 mAU in the wash phase. The AEX flow-through fraction was further purified by cation exchange (CEX) chromatography using CaptoSpImpRes resin (GE Healthcare, USA). The operating parameters used for this purification step are listed in Supplementary Table 1. The pooled eluent was analyzed for step recovery. The pooled eluent from CEX chromatography was filtered using Planova 20N virus removal filters as per manufacturer’s recommendations (Asahi Kasei, Japan) to remove residual virus particles, if any, from the anti-CD20 antibody. Finally, pooled filtrates were analyzed for anti-CD20 antibody recovery.

#### Diafiltration and ultrafiltration

The virus filtration output was subjected to diafiltration by buffer exchanging into final formulation buffer and to ultra-filtration for adjusting the final concentration of anti-CD20 antibody. The antibody was filtered using Pellicon 3, 30 kDa-Biomax tangential flow filtration (TFF) cassette (Merck Millipore, USA) as per manufacturer’s recommendations. Post-diafiltration, the anti-CD20 antibody was further concentrated to 10 mg/mL. The final TFF pool was analyzed for step recovery.

### Measurement of osmolality, metabolites, and antibody yield

Osmolality of the culture samples was measured using the Osmometry kit (Osmopro, Advanced Instruments, USA) as per manufacturer’s recommendations. The antibody yield was measured by its optical density and the metabolites were analyzed using a metabolite analyzer (Cedex Bio, Roche, Switzerland).

### Glycan analysis

Glycan analysis was performed to study the heterogeneity of the test anti-CD20 antibody sample (P2-CRD-1508) compared to that of the control antibody sample (RS-03-25), in terms of glycosylation, which is a common post-translation modification in recombinant monoclonal antibodies expressed in mammalian cell lines. Sample preparation and glycan labeling was done using the glycan labeling kit (Ludger, UK). Antibody samples were first reduced and alkylated. N-glycans were released from the Fc part of reduced antibody samples after digestion with PNGase enzyme. Released N-glycans were separated by using S-cartridges and concentrated by centrifugal evaporation. The dried N-glycan was derivatized and labeled using 2–AB (amino benzamine) florescent dye. Waters Acquity® liquid chromatography system (Waters, USA) with fluorescence detector (excitation, 330 nm; emission, 420 nm) was employed to separate and monitor 2-AB labeled glycans using Acquity BEH glycan column (Waters, USA) with a run time of 55 min in gradient elution with mobile phase-A (100 mM ammonium formate buffer, pH 4.5), mobile phase-B: (100% acetonitrile), and mobile phase-C (water for injection).

### Antibody-dependent cellular cytotoxicity (ADCC) assay

The ADCC activity of the test anti-CD20 antibody (P2-CRD-1508) was measured in comparison to that of the control antibody (RS-03-25). ADCC assay was performed using WIL2-S (ATCC, USA) as target cells and Jurkat/FcγRIIIa/NFAT-Luc cells (Promega, USA) as effector cells in the ratio of 1:7. The final concentrations of anti-CD20 antibody samples and control antibody samples in the wells, before adding the bio-glow reagent, were from 1 μg/mL to 0.0000038 μg/mL. ADCC activity by adding the test and control anti-CD20 mAbs to the WIL2-S as “target cells” and Jurkat T cells as “effector cells”. The crosslinking of mAb and FcγR will stimulate ADCC which is based on the binding affinities of this interaction. WIL2-S cell suspension was added into the positive control, and the sample, target control, and target plus effector control were added to the respective wells of a 96-well plate and incubated with effector cells for 5 h. After incubation, the Bio-glow Luciferase reagent (Promega, USA) was added to the positive control, sample, and assay control wells followed by incubation for 5 to 10 min at 25 °C. Finally, the luminescence was measured in the BioTek Synergy H1 plate reader (Biotek, USA) and the results were analyzed using customized PLA software. The differential affinities of Fc portions of test and control antibodies [due to their different fucose content] to crosslink to FcγR on effector cells lead to drastically different ADCC activities.

### Surface plasma resonance binding analysis

The binding affinity of the test anti-CD20 antibody (P2-CRD-1508) and the control antibody samples (RS-03-25) for FcγRIIIa receptors V158 and F158 (Acro Biosystems, USA) was determined by the SPR method using Biacore T200 (GE Healthcare, USA) as per manufacturer’s recommendations using immobilized anti-histidine CM5 sensor chip (GE healthcare, USA) and His-tagged FcγRIIIa receptors. The association (*k*_a_), dissociation (*k*_d_) constants, and binding affinity (*K*_D_) data were analyzed using Biacore T200 evaluation software. A total of six concentrations (500, 250, 125, 62.5, 31.25, and 15.6 nM) of the test and control antibody were run to determine the binding affinity.

## Results

### CHO clone development

**Fig. 2:**
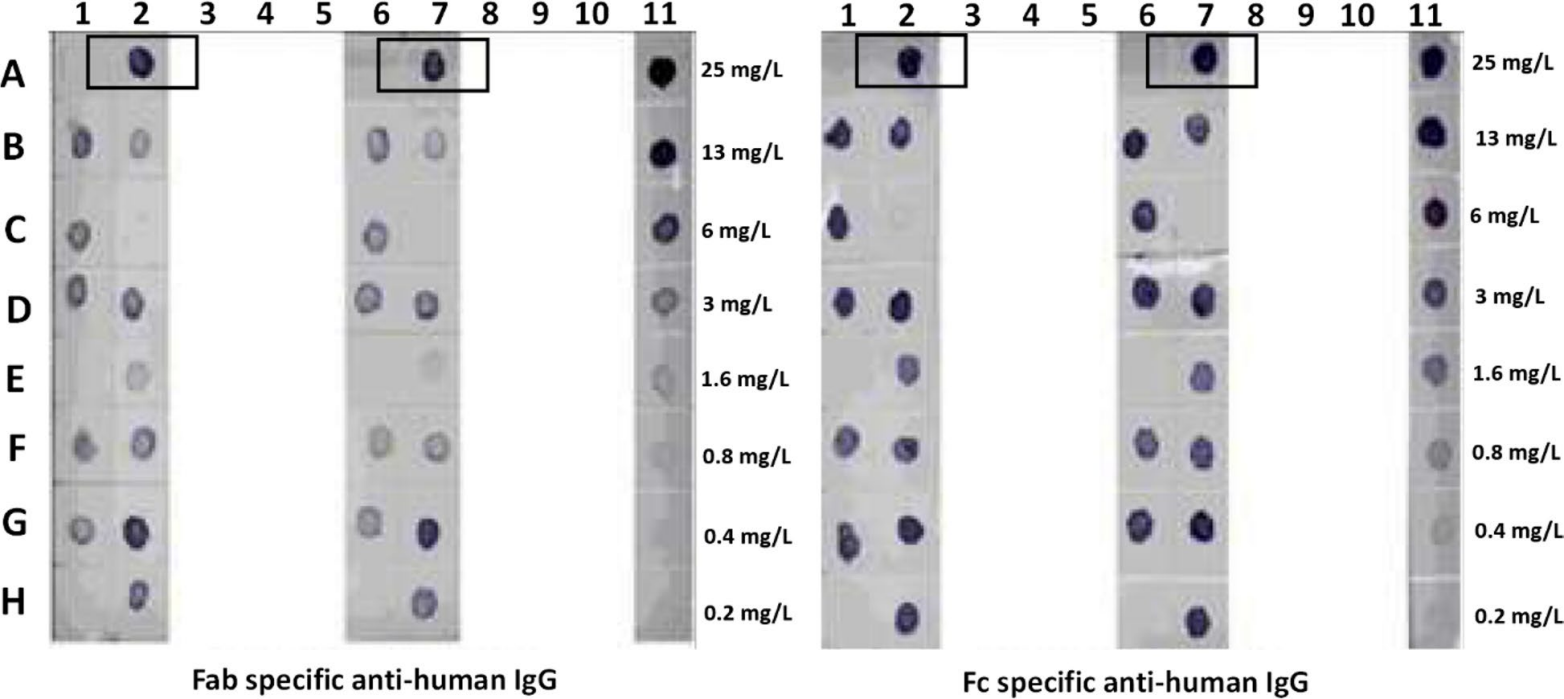
Dot blot analysis showing the best performing H3F9 (A2 and A7) clone that exhibited higher expression of anti-CD20 antibody was chosen and subcloned for shake flask culture

After screening all the double clones for growth and productivity using the dot-ELISA analysis (Supplementary Table 2 and Fig. 2) over a 14-day growth period, it was observed that the high performing cell line CHOSI Rit H3F9 produced 1.4 g of anti-CD20 mAb per liter. Hence, the H3F9 clone was selected for the expression of anti-CD20 mAb in the upstream cell culture experiments.

### Expression of anti-CD20 monoclonal antibody with higher afucosylation

The experimental cells were harvested on the 11th day, and the antibody productivity on day 11 was 1326 mg/L. The maximum viable cell count achieved was 3.628 × 10^7^ cells/mL on day 7 and cell viability was maintained above 95% until day 10 (Table 2). Lactate accumulation was high in the early exponential phase, but was later consumed by the growing cells (Table 2).

**Table 2:** Cell count and viability on different days of bioreactor process

Culture day	VCC (10^**6**^ cells/mL)	Viability (%)	Glucose (g/L)	Lactate (g/L)
0	0.56	99.6	9.53	0.19
1	1.16	99	8.26	0.27
2	2.66	97.9	8.17	0.98
3	6.18	98.4	7.55	1.68
4	19.75	99.3	7.23	1.99
5	25.93	99.03	6.81	2.37
6	34.28	98.8	6.27	2.98
7	36.28	97.8	6.17	2.19
8	33.87	98.8	5.88	1.65
9	32.71	96	5.29	1.08
10	34.52	97.1	4.64	0.94
11	30.18	96.7	3.25	0.53

The analysis of the afucose content in the anti-CD20 mAb expressed in the cells cultured with and without 8X uridine, manganese and galactose (UMG) showed that there was an over 2-fold increase (5.42 vs. 13.12). The osmolality of the medium decreased after the addition of 8X UMG from 440 mOsm to 326 mOsm (Table 3).

**Table 3:** Glycan data showing increase in afucose before and after addition of 8X UMG

Glycans (%)	Before adding 8X UMG	After adding 8X UMG
Total galactose	44.24	39.80
Total mannose	7.70	8.43
Total fucosylation	82.00	71.8
Total afucose	5.42	13.12
Osmolality on day 11	440 mOsm	326 mOsm

### Purification of anti-CD20 monoclonal antibody

#### Affinity chromatography

The step recovery in the affinity chromatography was observed to be 92%. Supplementary Figure S1 shows the chromatogram for affinity chromatography. It was observed that the MabSelectSuRe LX® resin with 50 mg/mL dynamic binding capacity at 6 min residence time could yield > 80% step recovery.

#### Anion exchange chromatography

The recovery from the anion exchange chromatography was estimated to be ~ 83%. Supplementary Figure S1 represents the anion exchange chromatography chromatogram. Q-Sepharose FF® resin with 60 mg of anti-CD20 antibody per mL of resin as load capacity at 8 min residence time was considered optimal for maximum product recovery and impurity removal.

### Cation exchange chromatography

The step recovery rate from the cation exchange chromatography was observed to be 79%. Supplementary Figure S1 shows chromatogram for the cation exchange chromatography from the confirmatory experiment. It was observed that Capto SP ImPres® resin with 50 mg anti-CD20 antibody per mL of resin as binding capacity at 6 min residence time with elution buffer of 50 mM Tris and 100 mM NaCl, at pH 9.0 was optimal.

### Nano filtration

The Planova 20N filter was used for nanofiltration of anti-CD20 mAb. The filtration details with flow rates are shown in **(**Supplementary Figure S2). The anti-CD20 mAb recovery was observed to be 100%.

### Diafiltration and ultrafiltration

During the diafiltration, the filtrate flux was observed as 80 LMH and there was no decrease in flux until the end of the diafiltration process. A flux drop was observed at final concentration. No product was observed in the permeate (waste flow). The Tangential flow filtration (TFF) output recovery was found to be 95%. Based on these observations, it was opined that Pellicon 3, 30 kDa-Biomax 30 kDa cassette with polyether sulfone membrane was suitable for the diafiltration and concentration of anti-CD20 antibody.

Overall antibody recovery from the final antibody purification process was 53%. The total purified antibody obtained was 706 mg from 1326 mg titer from one liter of cell culture supernatant. The purified anti-CD20 antibody (Sample Number: P2-CRD-1508) was used for further functional characterization.

### Glycan analysis

The test anti-CD20 antibody sample exhibited certain differences in the glycan profile when compared to control anti-CD20 antibody commercially available in the market (Supplementary Figure S3 and Table 4). Particularly, the afucosylation level of test anti-CD20 antibody was found to be over 10-fold higher when compared to that of the control antibody (Table 4). The levels of other glycan forms, such as galactose, mannose, and fucose also exhibited differences, but were not significant in terms of functional activity.

**Table 4:** Glycan values of test anti-CD20 antibody and control antibody

Glycan forms	Control antibody (RS-03-25)	Test anti-CD20 antibody (P2-CRD-1508)
Total galactose	51.1	39.80
Total mannose	2.6	8.43
Total fucose	88.9	71.8
Total afucose	1.2	13.12

### Antibody-dependent cellular cytotoxicity (ADCC) assay

**Fig. 3:**
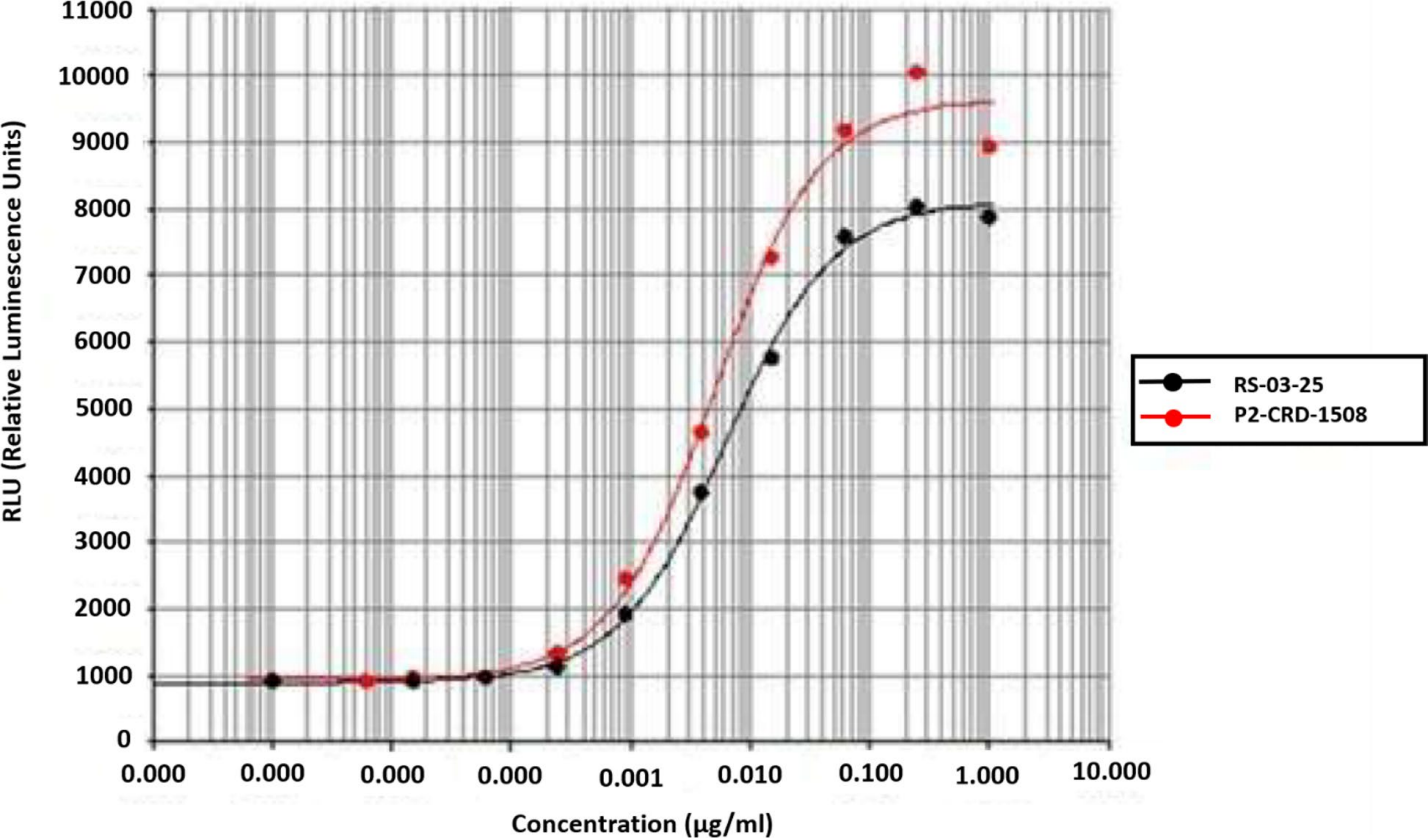
Dose response curve generated by parallel line analysis (PLA) for ADCC activity of test anti-CD20 antibody and control antibody. The test antibody P2-CRD-1508 showed higher ADCC activity due to increase in afucosylation (decrease level of fucosylation) over the control antibody (RS-03-25)

The test anti-CD20 antibody (P2-CRD-1508) showed over 2-fold higher (203%) ADCC activity when compared to the control antibody (RS-03-25) as shown in Fig. 3. The differential affinities of Fc portions of test and control antibodies to crosslink to FcγR on effector cells lead to drastically different ADCC activities. The higher ADCC activity of test anti-CD20 antibody over the control antibody is due to high afucosylation in the test antibody.

### Fc receptor-binding assay

**Fig. 4:**
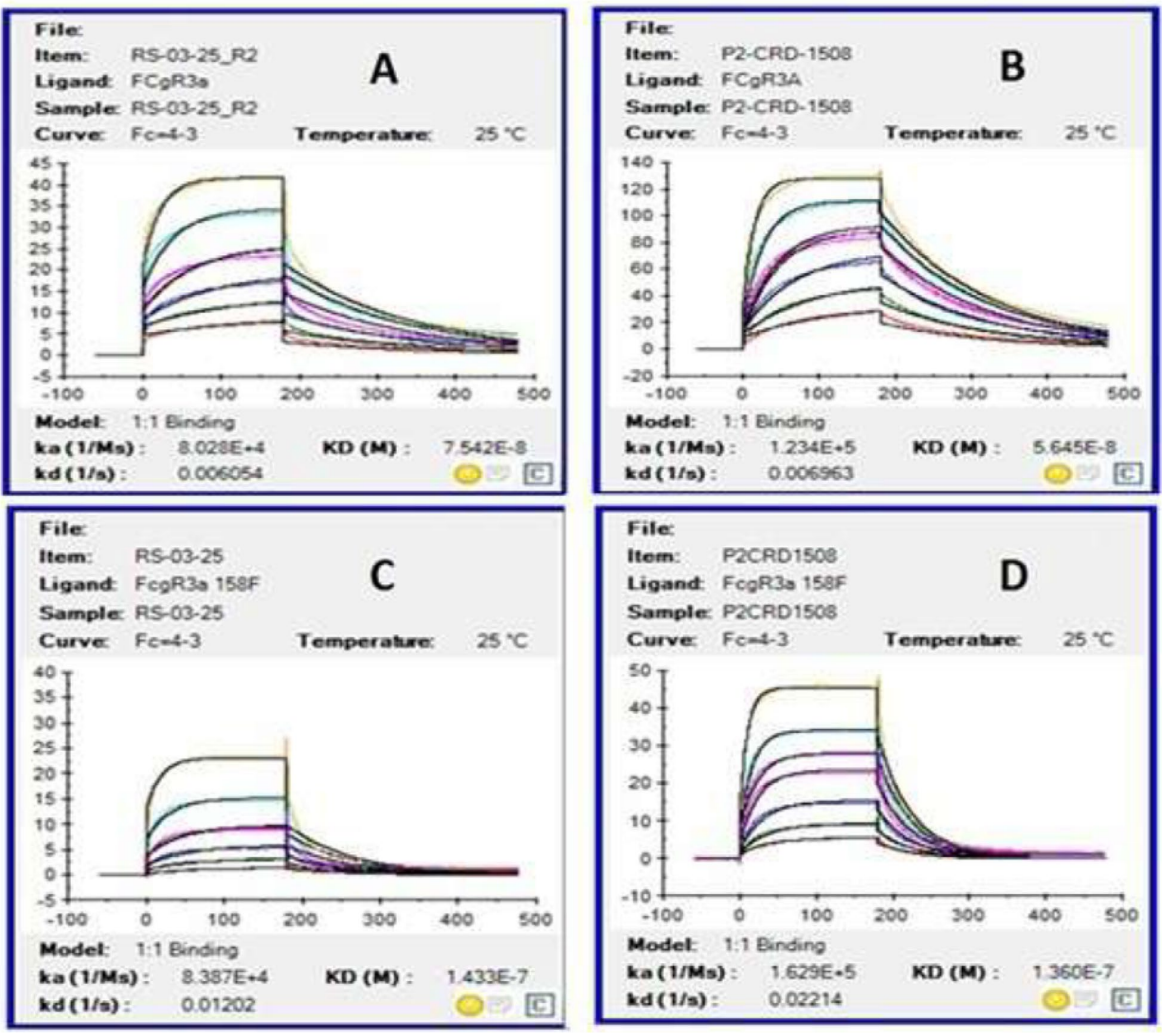
SPR sensorgrams obtained for FcγRIIIa V158 (**A**, **B**) and F158 (**C**, **D**) receptors for the test anti-CD20 antibody (P2-CRD-1508) (**B**, **D**) and control antibody (RS-03-25) (**A**, **C**). It can be seen from the figure that the association (*k*_a_), dissociation (*k*_d_), and binding affinity (KD) of test antibody (P2-CRD-1508) was high in comparison to control antibody (RS-03-25)

Binding affinity of the test anti-CD20 antibody (P2-CRD-1508) and control antibody (RS-03-25) for the FcγRIIIa receptors (V158 and F158) was determined by using surface plasma resonance (SPR). The association (*k*_a_), dissociation (*k*_d_) and binding affinity (*K*_D_) data showed that the binding affinity of the test anti-CD20 antibody to the FcγRIIIa V158 and F158 receptors (Fig. 4 and Table 5) was very high compared to that of the control antibody. High affinity to FcγRIIIaV158 and F158 is the mechanism behind increased ADCC activity of the test anti-CD20 antibody, which in turn was due to high afucosylation.

**Table 5:** SPR kinetic values obtained for FcγRIIIa V158 (A and B) and F158 (C and D) receptors for the test anti-CD20 antibody and control antibody

Receptor	Samples	***K***_**D**_ (nM)	***k***_**a**_ (1/Ms)	***k***_**d**_ (1/s)
FcγRIIIa V 158	Control antibody (RS-03-25)	75.4	8.028E + 04	0.006054
	Test antibody (P2-CRD-1508)	56.4	1.234E + 05	0.006963
FcγRIIIa F 158	Control antibody (RS-03-25)	143.3	8.387E + 04	0.01202
	Test antibody (P2-CRD-1508)	136.0	1.629E + 05	0.02214

## Discussion

Immunoglobulin G (IgG) consist of two heavy and two light chains. Both heavy chains contain three domains, CH1, CH2, and CH3, with a hinge region between CH1 and CH2. Additionally, the heavy chains contain biantennary N-glycan structures linked to Asn297 in the CH2 domain [15]. The N-glycan chains on each of the CH2 domains are often different, leading to asymmetrical binding of Fc to the Fc-receptors and thereby contribute to antibody effector functions [16–19]. The glycosylation on the Fc region also imparts stability to the Fc structure [20]. N-glycosylation is considered the most crucial post-translational modification that often results in a remarkable heterogeneity in antibody glycoforms [15]. Therapeutic antibodies expressed in mammalian cell expression systems generally have complex biantennary oligosaccharides in the Fc regions. The glycans of these antibodies constitute a core fucose and a bisecting N-acetyl glucosamine. In addition, they also vary in their terminal galactose and sialic acid content. An important Fc region mediated immune effector function that play prominent role in removal of tumor cells is antibody-dependent cell-mediated cytotoxicity. It is known that lack of core fucose on Fc N-glycan structure contributes to enhanced ADCC activity. Apart from afucosylation, galactosylation levels also influence the antibody effector functions such as CDC and ADCC. However, the role of afucosylation in modulation of these effector functions is much more prominent [21].

The therapeutic anti-CD20 mAbs also have a biantennary structure with a core fucose bound to the Fc region of the antibody. Anti-CD20 mAbs cause cell death through different pathways such as complement-dependent cytotoxicity (CDC), antibody-dependent cell-mediated cytotoxicity (ADCC), and apoptosis [22]. Suboptimal response and/or resistance to rituximab remain a challenge in the therapy of B cell non-Hodgkin’s lymphoma. Second-generation anti-CD20 antibodies focus mainly on increasing the CDC activity, while the third generation mAbs focus primarily on increasing the ADCC activity by engineering the antibody Fc portion to increase their binding affinity for the FcγRIIIa receptor [13]. Although these antibodies display increased ADCC activity, they have weaker CDC activity or vice versa. The glycoengineering process used for the generation of second and third generation antibodies is time consuming, tedious and needs more resources. In this study, we developed an anti-CD20 molecule with higher ADCC activity by simply modulating the fucose content of the antibody in CHO cell cultures using feed additives. The percentage of afucose is known to be the primary influencing factor for the binding of antibodies to the FcγRIIIa receptor, which in turn influences ADCC activity [23]. Afucosylation is a post-translational modification that does not require any modification in the genetic sequence. Modulating afucosylation in cell cultures is the cost effective and simple way to increase the ADCC activity and efficacy of the anti-CD20 monoclonal antibody. Previous reports have suggested a few approaches to increase afucosylation such as by overexpressing beta1-4-N-acetylglucosaminyltransferase III (GnTIII) enzyme, knocking out the endogenous FUT8 gene in CHO cells, and supplementing the media with fucosyltransferase inhibitors such as 2F-Peracetyl-fucose [24, 25]. However, genetic engineering-based approaches are tedious and require expensive resources.

Our approach to increase afucosylation by simple modulation of osmolality yielded higher ADCC activity and FcγRIIIa binding affinity in the test antibody compared to the commercially available anti-CD20 antibody. The percentage afucosylation was inversely correlated with osmolality, irrespective of the compounds used to adjust osmolality (ionic vs. non-ionic or substrate vs. non-substrate). The percentage of afucosylation can be controlled arbitrarily from 45 to 85% by feeding media with different osmolality (260–330 mOsm/kg) into cell cultures. There are reports regarding the effect of manganese on the afucosylation in cell cultures [10, 24]. These reports suggest that manganese has a dose-dependent effect on afucosylation, which in turn increases the affinity of the antibody to the FcγRIIIa receptors on effector cells. Since our objective is to increase afucosylation in order to increase ADCC activity of the antibody, we used different mix of feed additives to modulate osmolality and increase afucosylation. Results from the present study point toward control of both galactosylation and afucosylation of monoclonal antibodies produced from CHO cells lines can be influenced by UMG supplementation. A highly optimized concentration of feed additives such as uridine (8 mM), manganese ions (250 μM), and galactose (12.21 mM) control galactosylation and afucosylation levels of glycan structures. The final limits of these factors were set based on previous studies and our own preliminary studies on this antibody. These feed additives did not affect the culture performance, productivity nor the protein attributes of the resulting antibody when compared to the commercial antibody. Our results prove that osmolality is inversely proportional to afucosylation. The control experiment showed more osmolality before adding the feed additives compared to the batch after adding the feed additives on day 11. The percentage of afucosylation was almost double when compared to the control on day 11. The ADCC activity of the expressed anti-CD20 monoclonal antibody was found to be 203% higher compared to that of the control antibody.

## Conclusions

Introduction of anti-CD20 monoclonal antibody has changed the treatment options and improved survival of lymphoma patients. It is also the first mAb approved by FDA for treating the NHL. Glycoengineering of anti-CD20 mAbs through simple optimization of cell culture media components to obtain desired afucosylation has improved the natural effector functions of anti-CD20 mAb such as ADCC and CDC. However, the produced anti-CD20 antibody need to be further investigated for the efficacy in terms of ADCC activity in animal studies and human clinical trials. Combining the novel anti-CD20 mAb in radioimmunotherapy and CHOP regimen can provide the most promising treatment outcomes for aggressive NHL patients.

## Supplementary Information

**Additional file 1: Figure S1.** Chromatograms corresponding to affinity, anion exchange (AEX), and cation exchange (CEX) chromatography for purification of anti-CD20 antibody. Antibody recovery from each purification step was 92 %, 83 % and 79 % by affinity, anion exchange and cation exchange chromatography respectively. **Figure S2.** CEX output was subjected to filtration by Planova 20 N filter to remove any residual viral particles. The recovered output after filtration is 100 % with no loss. **Figure S3.** Overlaid glycan chromatogram profiles of test anti-CD20 antibody and control antibody. The levels of fucose is less in test antibody (P2-CRD-1508) in comparison to the control antibody (RS-02-25) which is responsible for enhanced ADCC activity of test antibody. **Supplementary Table 1.** Details of affinity, AEX and CEX chromatography phases, buffers, and operating parameters. **Supplementary Table 2.** Dot-blot sample scheme layout for Fig. 1.

## Data Availability

Raw data supporting the findings of this study are available with corresponding author (Vijaya R. Dirisala) and will be provided upon request.

## Abbreviations

ADCC: Antibody-dependent cellular cytotoxicity
CD16: Cluster of differentiation molecule 16 (FcγRIII)
CDC: Complement-dependent cytotoxicity
CH: Constant heavy chain
CHO: Chinese hamster ovary
CHOP: Cyclophosphamide, hydroxydaunorubicin, oncovin, prednisone
ESI-MS: Electrospray ionization-mass spectrometry
Fc: Fragment crystallizable
HC: Heavy chain
HTLV: Human T cell lymphotropic virus
IgG: Immunoglobulin G
LC: Light chain
mAb: Monoclonal antibody
N-glycan: Amino-linked glycan
PNGase: Peptide-N-glycosidase
UMG: Uridine, manganese chloride, galactose
