# Supplementary material for: Enhancement of effector functions of anti-CD20 monoclonal antibody by increased afucosylation in CHO cell line through cell culture medium optimization
Source: J Genet Eng Biotechnol. 2022 Oct 4;20:141. doi: 10.1186/s43141-022-00421-5 (PMC9532503; doi:10.1186/s43141-022-00421-5)
Supplement: Supplementary file 1 — Additional file 1: Figure S1. Chromatograms corresponding to affinity, anion exchange (AEX), and cation exchange (CEX) chromatography for purification of anti-CD20 antibody. Antibody recovery from each purification step was 92 %, 83 % and 79 % by affinity, anion exchange and cation exchange chromatography respectively. Figure S2. CEX output was subjected to filtration by Planova 20 N filter to remove any residual viral particles. The recovered output after filtration is 100 % with no loss. Figure S3. Overlaid glycan chromatogram profiles of test anti-CD20 antibody and control antibody. The levels of fucose is less in test antibody (P2-CRD-1508) in comparison to the control antibody (RS-02-25) which is responsible for enhanced ADCC activity of test antibody. Supplementary Table 1. Details of affinity, AEX and CEX chromatography phases, buffers, and operating parameters. Supplementary Table 2. Dot-blot sample scheme layout for Fig. 1. [file 43141_2022_421_MOESM1_ESM.docx]

**SUPPLEMENTARY FIGURES**

**Supplementary Fig. 1**


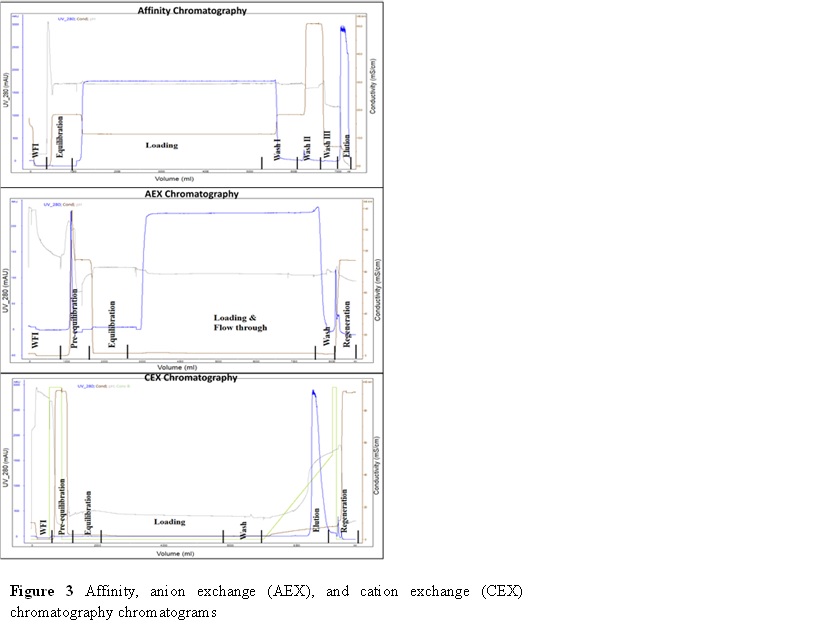


**Supplementary Fig. 2**


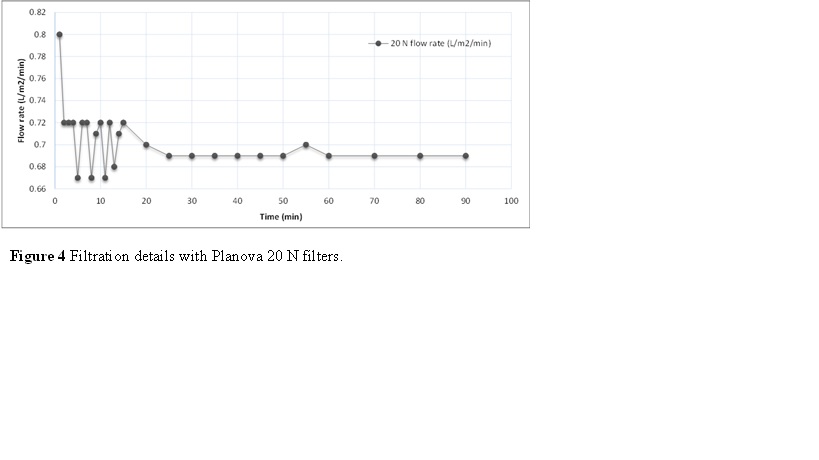


**Supplementary Fig. 3**


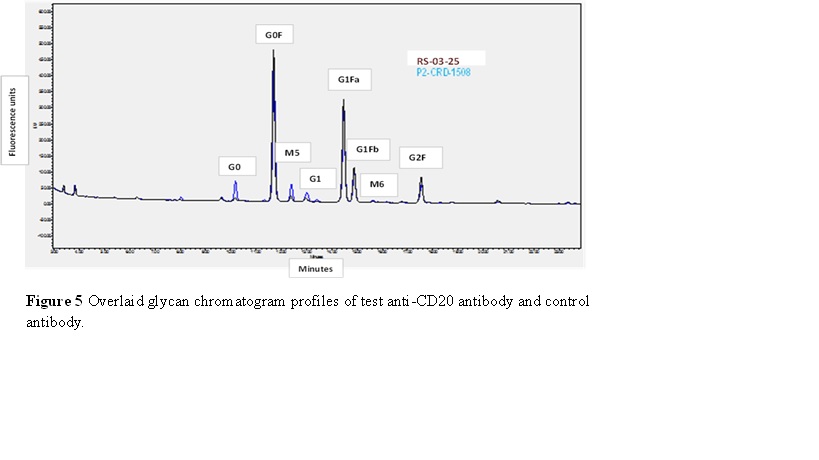


**Supplementary Table 1** Details of affinity, AEX and CEX chromatography phases, buffers, and operating parameters

| **Phase** | **Buffers** | **Volume (CV)** | **Flow rate (cm/h)** | **Residence time (min)** |
| --- | --- | --- | --- | --- |
| **Affinity Chromatography** | | | | |
| Equilibration | Affinity chromatography equilibration buffer | 5 | 200 | 6 |
| Loading | Affinity load (cell culture supernatant containing anti-CD20 antibody) | As per resin | 200 | 6 |
| Wash-I | Affinity chromatography equilibration buffer | 5 | 200 | 6 |
| Wash-II | Affinity chromatography wash-II buffer | 3 | 200 | 6 |
| Wash-III | Affinity chromatography wash-III buffer | 3 | 200 | 6 |
| Elution | Affinity chromatography elution buffer | 5 | 200 | 6 |
| **AEX Chromatography** | | | | |
| Equilibration | Anion exchange equilibration buffer | 5 | 200 | 6 |
| Loading | Anion exchange load | As per condition | 200 | 6 |
| Post loading wash | Anion exchange equilibration buffer | 5 | 200 | 6 |
| **CEX Chromatography** | | | | |
| Equilibration | Cation exchange equilibration buffer | 5 | 200 | 6 |
| Sample loading | Cation exchange load | 50 mg/ml of resin | 200 | 6 |
| Post loading wash | Cation exchange equilibration buffer | 3 | 200 | 6 |
| Elution | Cation exchange equilibration buffer & Cation exchange elution buffer | 3 | 200 | 6 |

**Supplementary Table 2** Dot-blot sample scheme layout for Fig. 1

|  | **1** | **2** | **3** | **4** | **5** | **6** | **7** | **8** | **9** | **10** | **11** |
| --- | --- | --- | --- | --- | --- | --- | --- | --- | --- | --- | --- |
| **A** | **Split 2, cd 15** | | | | | **Split 3, cd 08** | | | | |  |
|  | H3C3 | H3F9 | Empty | Empty | Empty | H3C3 | H3F9 | Empty | Empty | Empty | Std. 25 mg/L |
| **B** | H3C5 | H3F10 | Empty | Empty | Empty | H3C5 | H3F10 | Empty | Empty | Empty | Std.13 mg/L |
| **C** | H3C6 | XXX | Empty | Empty | Empty | H3C6 | XXX | Empty | Empty | Empty | Std.6 mg/L |
| **D** | H3D4 | H3G7 | Empty | Empty | Empty | H3D4 | H3G7 | Empty | Empty | Empty | Std.3 mg/L |
| **E** | H3D9 | H3G8 | Empty | Empty | Empty | H3D9 | H3G8 | Empty | Empty | Empty | Std. 1.6 mg/L |
| **F** | H3E8 | H3G11 | Empty | Empty | Empty | H3E8 | H3 G11 | Empty | Empty | Empty | Std.0.8 mg/L |
| **G** | H3E10 | H3 G12 | Empty | Empty | Empty | H3E10 | H3 G12 | Empty | Empty | Empty | Std. 0.4 mg/L |
| **H** | H3F6 | H3H8 | Empty | Empty | Empty | H3F6 | H3H8 | Empty | Empty | Empty | Std. 0.2 mg/L |
